# The Dance of Tusks: Rediscovery of Lower Incisors in the Pan-American Proboscidean *Cuvieronius hyodon* Revises Incisor Evolution in Elephantimorpha

**DOI:** 10.1371/journal.pone.0147009

**Published:** 2016-01-12

**Authors:** Dimila Mothé, Marco P. Ferretti, Leonardo S. Avilla

**Affiliations:** 1 Laboratório de Mastozoologia, Departamento de Zoologia, Instituto de Biociências, Universidade Federal do Estado do Rio de Janeiro, Av. Pasteur, 458, 501, Urca, CEP 22290–240, Rio de Janeiro, Brazil; 2 Programa de Pós-graduação em Ciências Biológicas (Zoologia), Museu Nacional/UFRJ, Quinta da Boa Vista, CEP 20940–040, Rio de Janeiro, Brazil; 3 Dipartimento di Scienze della Terra e Museo di Storia Naturale, Sez. di Geologia e Paleontologia, Università di Firenze, via G. La Pira 4, I-50121 Firenze, Italy; 4 Programa de Pós-graduação em Biodiversidade Neotropical, Instituto de Biociências, Universidade Federal do Estado do Rio de Janeiro, Av. Pasteur, 458, 501, Urca, CEP 22290–240, Rio de Janeiro, Brazil; New York Institute of Technology College of Osteopathic Medicine, UNITED STATES

## Abstract

The incisors of proboscideans (tusks and tushes) are one of the most important feature in conservation, ecology and evolutionary history of these mammals. Although the absence of upper incisors is rare in proboscideans (occurring only in deinotheres), the independent losses of lower incisors are recognized for most of its lineages (dibelodont condition). The presence of lower incisors in the Pan-American gomphothere *Cuvieronius hyodon* was reported a few times in literature, but it was neglected in systematic studies. We analyzed several specimens of *Cuvieronius hyodon* from the Americas and recognized that immature individuals had lower incisors during very early post-natal developmental stages. Subsequently, these are lost and lower incisors alveoli close during later developmental stages, before maturity. Moreover, for the first time in a formal cladistic analysis of non-amebelodontine trilophodont gomphotheres, *Rhynchotherium* and *Cuvieronius* were recovered as sister-taxa. Among several non-ambiguous synapomorphies, the presence of lower incisors diagnoses this clade. We recognize that the presence of lower incisors in *Cuvieronius* and *Rhynchotherium* is an unique case of taxic atavism among the Elephantimorpha, since these structures are lost at the origin of the ingroup. The rediscovery of the lower incisors in *Cuvieronius hyodon*, their ontogenetic interpretation and the inclusion of this feature in a revised phylogenetic analysis of trilophodont gomphotheres brought a better understanding for the evolutionary history of these proboscideans.

## Introduction

The Pan-American proboscidean *Cuvieronius hyodon* (Fischer de Waldheim, 1814) is the only gomphothere known throughout the New World from the early to late Pleistocene [1–3]. Traditionally, it is stated that South American gomphotheres (*Notiomastodon platensis* and *Cuvieronius hyodon*, *sensu* [3,4]) lack lower defenses (both incisors, the deciduous—di1 or tushes—and the permanents—i1 or tusks) [5], such as observed in mammoths and extant elephants [6]. Accordingly, the dental formula of *C*. *hyodon* consists of one pair of upper tushes (deciduous incisors), one pair of upper tusks (evergrowing permanent incisors), three pairs of lower and upper deciduous premolars (first bilophodont—dp2/dP2—and the other two trilophodonts—dp3/dP3and dp4/dP4) and three pairs of upper and lower permanent molars (first two trilophodonts—m1/M1 and m2/M2 and last one tetra or pentalophodonts—m3/M3 [3]). The horizontal replacement of the deciduous premolars and permanent molars, together with the progressive wear on these teeth occlusal surface generate a wearing pattern that can be associated with developmental stages of the proboscidean (Table 1 [7–9]).

**Table 1:** Developmental stages and age classes for immature individuals of *Cuvieronius hyodon* based on lower tooth eruption, wear stage and estimated age in years (modified from [7,8]).

Developmental stage	dp2	dp3	dp4	m1	Age (in years)
**Fetus**	0/0+	0	-	-	0
**J1**	0+/1	0+/1	-	-	0.5
**J2**	2	1	-	-	0.5
**J3**	2/3	1/2	Forming	-	1
**J4**	3	2	0	-	1.5
**J5**	4	3	0/0+	-	2/2.5
**Y1**	Absent	3/4	1	Forming	3
**Y2**	Absent	4	2	0	4/5
**Y3**	Absent	Absent	3	0+/1	5/9
**Y4**	Absent	Absent	4	2	9/14
**Adult**	Absent	Absent	Absent	3	14/death

Teeth wear stages follow these symbols: 0 (crown formed but not fully erupted); 0+ (crown fully erupted but unworn);1 (wear on protoloph/id only); 2 (wear on all lophs/ids); 3 (extensive wear and all lophs/ids with adjacent enamel figures) and 4 (severe wear, enamel figures contiguous).

However, some authors reported the presence of lower incisors in two juvenile specimens of *C*. *hyodon* from Tarija, Bolivia [10–12]. Also, a mandible of another juvenile of *C*. *hyodon* with a pair of lower incisor alveoli was recognized from Costa Rica [13]. Additionally, the lower incisors of *C*. *hyodon* were considered as vestigial lower tushes [14], because these teeth are present only in a few immature individuals, whereas in most juvenile mandibles there are no tushes nor detectable traces of lower incisor alveoli. No further detailed morphological study on the lower incisors of *Cuvieronius hyodon* has been made since those reports, although this taxon has been widely studied in the last decades [1,3,14–21].

As a result, all subsequent phylogenetic analyses including this Pan-American gomphothere have neglected the observations of those authors [12–14], by neither considering *Cuvieronius hyodon* as having lower incisors, nor rating this taxon as polymorphic for this particular feature [1,5,6,21,22]. Here, we recognize ontogenetic and evolutionary patterns for the lower incisors, deciduous premolars and molars, and lower jaw of *Cuvieronius hyodon*. In addition, we present a phylogenetic hypothesis to non-amebelodontine trilophodont gomphotheres with emphasis in the position of *C*. *hyodon*. Moreover, we discuss the significance of the lower incisors features to the evolution of non-amebelodontine trilophodont gomphotheres and also to Elephantimorpha broadly speaking (*sensu* [23]).

## Results and Discussion

### The lower incisors of *Cuvieronius hyodon*

**Fig 1:**
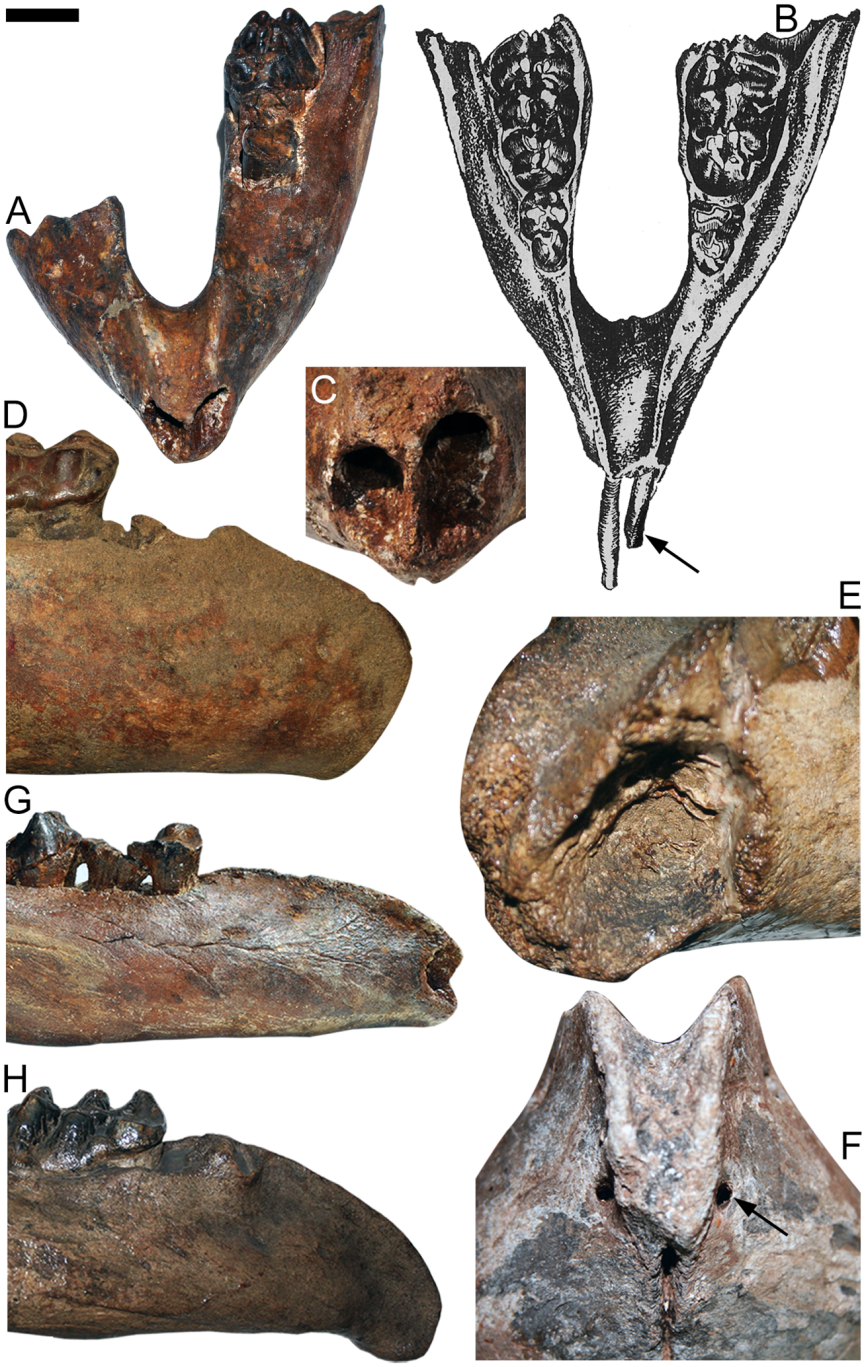
Juvenile mandibles of *Cuvieronius hyodon*. (A) Specimen MNPA-V 005888 with open lower tusk alveoli, from Tarija, Bolivia; (B) Schematic drawing of the specimen MNPA-V005867, bearing the pair of lower tusks, modified from [12]; (C) Detail of the same specimen in A, showing the open alveoli for the lower tusks; (D) Specimen MACN-Pv 12521, with no lower tusk alveoli, from Tarija, Bolivia; (E) Detail of the symphysis of the specimen TAR 807, showing a pair of closing lower tusk alveoli, from Tarija, Bolivia; (F) Detail of the symphysis of specimen UCR (uncatalogued), showing the closing pair of lower tusk alveoli, from Costa Rica; (G) Same specimen in A, with straight symphysis. (H) Specimen TAR 806, with downturned symphysis, from Tarija, Bolivia. The arrow in (B) indicates the lower tusks and the enamel band on left lower tusk; the arrow in F indicates the pair of closing lower alveoli. Scale bar = 5 cm.

**Fig 2:**
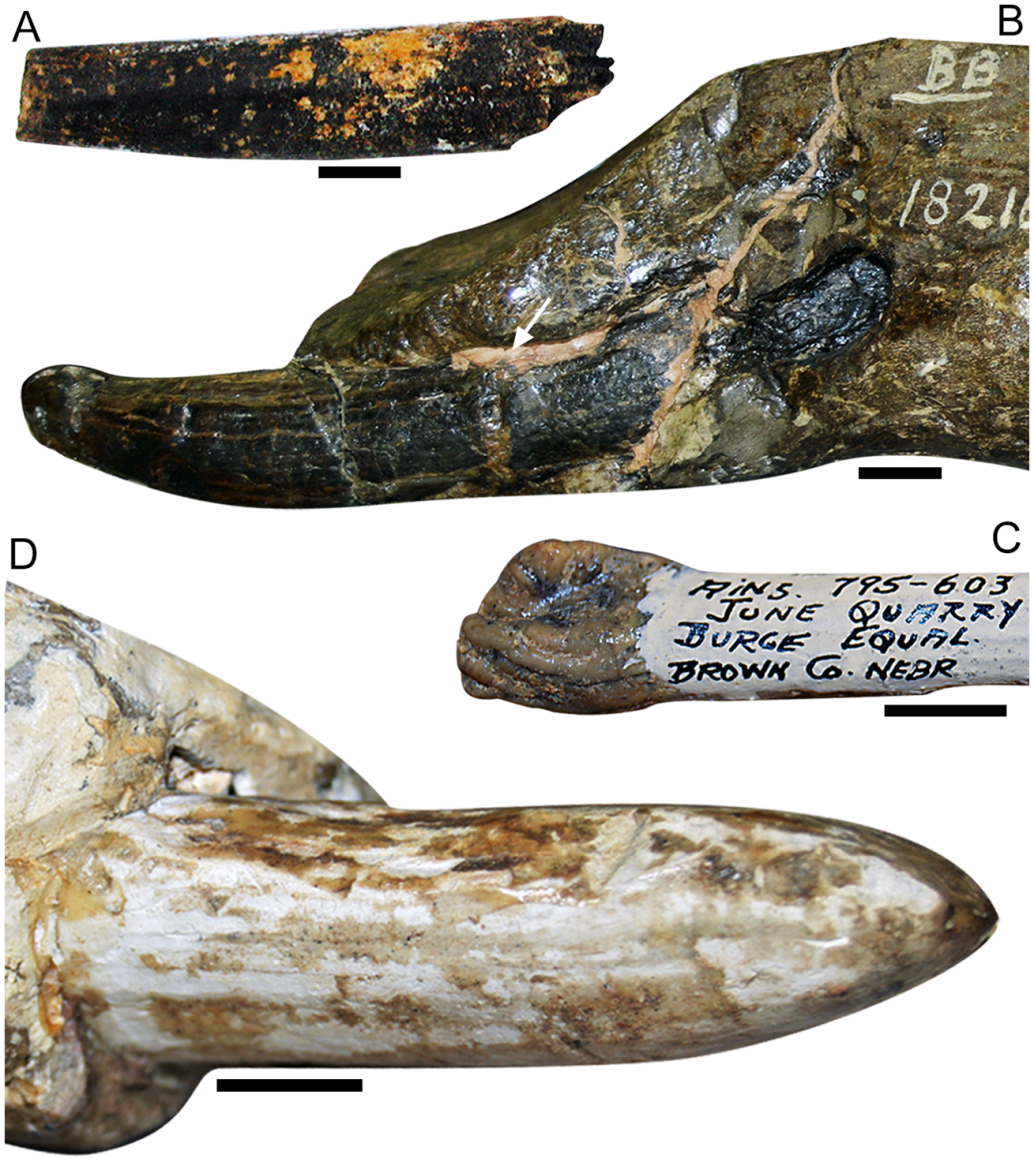
Lower tusks of *Cuvieronius*, *Rhynchotherium* and *Gomphotherium*. (A) Radical fragment of a right lower tusk (MNPA-V 005867) of *C*. *hyodon* from Tarija. This is the sole recent picture (taken in 2003) of a lower tusk of *Cuvieronius hyodon*. The specimen is at present (2015) missing. (B) Juvenile specimen of *Rhynchotherium*, F:A.M. 18216, with small lower tusks, from California, USA; (C) Lower deciduous tusk of new born *Gomphotherium*, (uncatalogued), from Nebraska, USA. Note the slightly inflated and wrinkled enamel cap. (D) Lower tusks of a juvenile *Gomphotherium*, specimen F:A.M. 21112, from Santa Fé, New Mexico, USA. The arrows indicate the enamel band on the lower tusks. Scale bar = 1 cm.

Among 46 individuals of *C*. *hyodon* analyzed, we identified seventeen individuals (eleven juveniles and six adults) with lower incisor alveoli (approximately 37% of the sample analyzed, see Table 2, Fig 1A and 1D). Two mandibles with lower incisors preserved the lower incisors *in loci* [12, 24] (Fig 1B). Unfortunately, these specimens were damaged and the lower incisors were lost, probably during the transfer from the Museo de Historia Natural de Bolivia (La Paz) to the Museo Nacional de Paleontología y Arqueología de Tarija (Fig 1C). A small fragment of the right incisor was still preserved in mandible MNPA-V 005867 (Fig 2A) when one of the present authors visited the Tarija Museum in 2003.

**Table 2:** Analyzed mandibles of *Cuvieronius hyodon*, their developmental stage, the presence/absence of lower incisors, and shape of mandibular symphysis.

Specimen	Developmental stage	Lower incisor	Symphysis shape
**MNPA-V 255**	J1	Open alveoli	Downturned
**TAR 1234**	J1	Broken	Broken
**MNPA-V 005867**	J3	Open alveoli	Straight
**MNPA-V 005865**	J4	Open alveoli	Straight
**MACN 1500**	J4	Broken	Broken
**NMR 4427**	J5	Open alveoli	Straight
**MNPA-V 005869**	J5	Open alveoli	Downturned
**MNPA-V 005888**	J5	Open alveoli	Straight
**MACN 12567**	Y1	No trace of alveoli	Straight
**MNPA-V 005871**	Y1	Broken	Straight
**MNPA-V 92**	Y1	No trace of alveoli	Straight
**MNPA-V 006140**	Y2	No trace of alveoli	Downturned
**TAR 807**	Y2	Closing alveoli	Straight
**TAR 806**	Y2	No trace of alveoli	Downturned
**AMNH-DVP 101395**	Y2	Closing alveoli	Downturned
**TAR 805**	Y2	Closing alveoli	Straight
**MUT-V 33**	Y3	No trace of alveoli	Straight
**MNPA-V 006330**	Y3	No trace of alveoli	Downturned
**UCR uncatalogued**	Y3	Closing alveoli	Straight
**INAH uncatalogued**	Y3	Closing alveoli	Straight
**MNPA-V 006141**	Y3	No trace of alveoli	Downturned
**MUSM uncatalogued**	Adult	Closing alveoli	Straight
**MACN-PV 1292**	Adult	Closing alveoli	Downturned
**MACN-PV 1891**	Adult	Closing alveoli	Straight
**MACN-Pv 506**	Adult	Closing alveoli	Downturned
**MNPA-V 006194**	Adult	Closing alveoli	Downturned
**NMR 7783**	Adult	Closing alveoli	Downturned
**MNPA-V 006125**	Adult	No trace of alveoli	Downturned
**MACN 509**	Adult	No trace of alveoli	Straight
**MACN 599**	Adult	No trace of alveoli	Straight
**MACN 601**	Adult	No trace of alveoli	Straight
**MACN-PV 14639**	Adult	No trace of alveoli	Straight
**MNPA-V 006184**	Adult	No trace of alveoli	Straight
**MNPA-V 005806**	Adult	No trace of alveoli	Straight
**MNPA-V 006125**	Adult	No trace of alveoli	Downturned
**MNPA-V 006152**	Adult	No trace of alveoli	Downturned
**TAR 1270**	Adult	No trace of alveoli	Straight
**MACN-Pv 502**	Adult	No trace of alveoli	Straight
**MACN-Pv 508**	Adult	No trace of alveoli	Straight
**MACN-PV 600**	Adult	Broken	Straight
**MACN-Pv 602**	Adult	No trace of alveoli	Straight
**MNPA-V 005804**	Adult	No trace of alveoli	Straight
**MNPA-V 005845**	Adult	No trace of alveoli	Downturned
**AMNH-DVP 26985**	Adult	No trace of alveoli	Straight
**MNPA-V 005805**	Adult	No trace of alveoli	Straight

The lower incisors of *Cuvieronius* are small, well-preserved and containing enamel [12]; and two mandible specimens were figured (MNPA-V 005867 and MNPA-V 005888) with a pair of elongated, thin, parallel with convergent tips incisors (Figs 1B and 2A). Besides the morphological description of the lower incisors [12], a picture of specimen MNPA-V 005867 including a small lower incisor is the only direct record of this tooth (Fig 2A). Unlike the upper permanent tusks, the lower incisor of *C*. *hyodon* is elongated and thin, with 11 mm at its widest diameter, straight and not twisted (see Figs 1B and 2A). According with the shape of the lower alveoli, we infer that the lower incisor probably had an oval/rounded cross section (Fig 1A–1C). The presence of enamel in the lower incisor of *Cuvieronius*, is dubious: these teeth were described as clearly having enamel [12], however, no trace of enamel was noted by the only present author that visited the Tarija Museum before the incisor get lost (MPF). The proximal end of the incisor (pulp cavity) possessed and open pulp, although of limited size, which indicates that these incisors were evergrowing.

In fact, newly born and immature individuals of Gomphotheriidae are rare, especially those with complete deciduous dentition. *Rhynchotherium* is known only from a few specimens ([25] Fig 2B), whereas *Cuvieronius* has a more significant fossil record from Bolivia and Mexico [11,12,26,27]. One of the youngest records of immature *Cuvieronius* is a newborn skull specimen from Lake Chapala, Jalisco, Mexico, which presents the upper tushes *in loci* [26]. The upper tushes are quite similar to the upper tushes of *Gomphotherium angustidens* [28], since both are small, conical, with pointed and slightly wrinkled enamel collar.

However, concerning immature individuals, *Gomphotherium angustidens* has a more complete fossil record than *Cuvieronius* and *Rhynchotherium*, which includes several specimens of tushes (deciduous incisors or ‘‘milk tusks”), tusks (the evergrowing permanent incisors), deciduous premolars, permanent premolars and molars [9,25,28–30]. *Rhynchotherium* is known only from one immature specimen (F:A.M. 18216) with deciduous premolars in use and erupted lower incisors. The lower incisors are slightly upcurved, coniform, not twisted; with a small lateral enamel band and oval cross section (see Fig 2A and 2B). Later in ontogeny, the lower incisors of *Rhynchotherium* become slightly upcurved and may lose their lateral enamel band. The presence of enamel bands on lower incisors of *Rhynchotherium* specimens is usually not easily observed [31]. However, the immature lower incisors of *Rhynchotherium* (Fig 2B) present this enamel structure, which generally is lost with usage and/or during ontogenetic development.

The lower tushes of *Gomphotherium angustidens* are represented by the di1 [9] and are small, straight, with slightly inflated apices, and piriform cross section (Fig 2C [28]). The apex of each tush is covered by an enamel cap, which is flattened, wrinkled at the tip and presents well-defined borders (“collar”, according to [9], Fig 2C). On the other hand, the immature lower tusks (i1) have pointed apices, with bean-shaped cross sections and a uniform enamel cap, which has no collar or well-defined borders [28]. As the individual grows, the enamel cap wears away, the tusks become longer and dorsoventrally flattened, and develop a piriform cross section [25,28] (see Fig 2D).

The lower tushes of *Gomphotherium angustidens* are very particular and only few immature individuals have these teeth preserved *in loci* [28]. The tissues responsible for the attachment of the lower incisors in the alveoli are very soft, fragile and they could deteriorate easily, thus causing the lower incisors to detach from the alveoli before the final burial of the mandible [30]. Therefore, loss of the lower incisors before fossilization is more likely to be a common pattern among gomphotheres, and the presence of open alveoli is the only evidence of the existence of these teeth.

### Growth and ontogeny of lower incisors in *Cuvieronius hyodon*

The developmental stages recognized for *Cuvieronius hyodon* in the sample analyzed here were J1, J3, J4, J5, Y1, Y2, Y3 and Adult. We did not recognized individuals in the developmental stages of Fetus, J2 and Y4, because none of those specimens presented the deciduous molars with the combination of wear pattern of these developmental stages (Table 1). There are 21 juvenile specimens between J1 and Y3, and 25 Adult specimens (Table 2). It is important to note that among juveniles between developmental stages J1 to J5 in which it was possible to observe the symphysial region, the frequency of lower incisor alveoli is 100% (i.e., all J1 to J5 specimens presented open lower incisor alveoli). Concerning the specimens on developmental stages Y1 to Y3, five specimens presented closing lower incisor alveoli (38.5% of Y1 to Y3 juveniles) and seven presented no trace of alveoli.

Among the Adults with preserved symphysis (24 individuals), only six specimens presented closing alveoli (25% of Adults), whereas 24 presented no trace of alveoli (Table 2). Therefore, we observed that until the developmental stage J5, all studied individuals of *Cuvieronius* carried lower incisors before death (except for two with broken symphysial region), suggesting their lower tusks were still being used before death, and the subsequent loss of these incisors was due taphonomic processes [9]. In the eleven individuals from Y1 to Adult which lower incisors alveoli, these structures were partially closing (i.e., filled with spongy tissue, shallow and usually reduced in size—Fig 1D and 1E).

It is possible to infer that, in the ontogenetic development of *C*. *hyodon*, the lower tusks were still being used until the developmental stage J5. These were subsequently lost around the developmental stage Y2, and the alveoli started to heal/close from this stage onwards. No individuals of stage Y1 present the symphysial region bearing lower incisor alveoli, thus, it was not possible to precisely infer the status of the lower incisors and lower incisors alveoli in this age group.

In other non-amebelodontine trilophodont gomphotheres with lower incisors, the youngest specimen of *Rhynchotherium* (F:A.M. 18216) has erupted lower incisors and with worn tips at the stage Y1, whereas the lower incisors of most immature *Cuvieronius* are probably lost at this developmental stage. At the stage Y3, the lower tusks of *Rhynchotherium* are longer, slightly upcurved and with divergent tips, following the downturned curvature of the mandibular symphysis. In *Gomphotherium angustidens*, the lower tushes are probably formed/erupted before the stage J1 stage, when both dp2 and dp3 become erupt [28]. The lower tushes are replaced by the lower tusks probably at the stage J2, when the dp2 and dp3 are erupted and slightly worn. As soon as they erupt, the lower tusks also become worn rapidly, and the small enamel cap at their tips wears away [25,28,32,33]. *Cuvieronius* individuals at J1 developmental stage already present a large open alveoli, which indicates the presence of completely erupted lower incisors. Probably, the lower incisors develop and erupt at the Fetus and J1 developmental stages and there is no evidence of replacement of the lower incisors until the stage J5. In addition, the absence of isolated lower incisors of *Cuvieronius* in the fossil record could be also an evidence of the no replacement of the lower incisor in *Cuvieronius*.

In addition, the brevirostrine non-amebelodontine trilophodont gomphotheres do not develop second generation lower teeth, as *Gomphotherium* had true permanent pre-molars and lower tusks [9,25,28]. The dentition of *Notiomastodon*, *Sinomastodon* and *Stegomastodon* comprises the upper pair of tushes, the upper and lower deciduous pre-molars and permanent molars, which derived from primary lamina. The upper tusks (I2) are the only teeth originated on the secondary lamina, which replaces the upper tushes in immature individuals (around J3 developmental stage). Furthermore, *Rhynchotherium* and *Cuvieronius* present the lower incisors, which are not replaced during lifespan. Probably, except for the presence of the I2, the secondary lamina was lost in the brevirostrine non-amebelodontine trilophodont gomphotheres. Thus, considering the eruption of lower incisor at Fetus/J1 developmental stage and the absence of replacement by the permanent lower incisors in *Cuvieronius*, we believe that is more parsimonious to consider its lower incisors as tushes (di1), developed from the primary lamina.

Usually, in mammals, the secondary generation (permanent) teeth develop only after the first generation (deciduous) teeth [34,35]. In the case of brevirostrine non-amebelodontine trilophodont gomphotheres, we believe that the secondary lamina was suppressed and the incisors of *Cuvieronius* are originated in the primary lamina, with no replacement. In fact, *Cuvieronius* retained in the mandible a pair of deciduous incisors, the deciduous premolars and molars, all originated in first generation. This "replacement pattern” (a modified “monophyodont" dentition, since there is no replacement) is similar to the one found in Murid rodents, which have eliminated primary and secondary generations of canines and premolars, and retained only a pair of deciduous incisors and the molars, with no dental replacement at all [36]. Since there is also no record of isolated incisors recognized as *Rhynchotherium*, and the fossil record does not present evidence of incisors replacement, probably the same inference can be made to this taxon.

The lower incisors of *C*. *hyodon* were considered as vestigial because they are present only in a few specimens of Tarija (Bolivia) [14]. However, the definition of a vestigial structure [37] is: 1) it occurs regularly in all members of a population; 2) it is present in the parents and recent ancestors; and, 3) it either occurs transiently during development or may persist into maturity (depending on the degree of evolutionary suppression). In this way, the presence of lower tushes in *Cuvieronius hyodon* is not vestigial. The argument presented by that author [14] better fits the evolutionary process of spontaneous atavism, in which rare atavistic anomalies occur in individuals of natural populations [38]. Nevertheless, we identified the presence of incisors in other populations of *C*. *hyodon* from Central and North America, and that persistent lower tushes are characteristic of the developmental stages J1 to J5, which correspond to the juvenile age class. Yet, the lower tushes of *C*. *hyodon* fails in two of the four criteria created to identify atavism [39]–the persistence of a feature in adults and the presence in only one or a few individuals within a population. For this reason, we agree with the vestigial presence of lower tushes in *C*. *hyodon*, but not for the same arguments provided by this author [14].

### Phylogenetic position of *Cuvieronius hyodon*

As previously mentioned, most cladistics analysis of non-amebelodontine trilophodont gomphotheres have not considered the presence of lower tusks in *Cuvieronius* [1,5,6,21,22]. Regardless of the particular taxonomic assumptions for the South American endemic gomphothere (*Notiomastodon* in [6], *Stegomastodon platensis* and *S*. *waringi* in [5], *Haplomastodon chimborazi* and “*S*.”*platensis* in [1], “*S*.” *waringi* and “*S*.” *platensis* in [22], and *Notiomastodon platensis* in [21]), all those previous phylogenetic hypotheses recovered *Cuvieronius* as its sister taxon.

**Fig 3:**
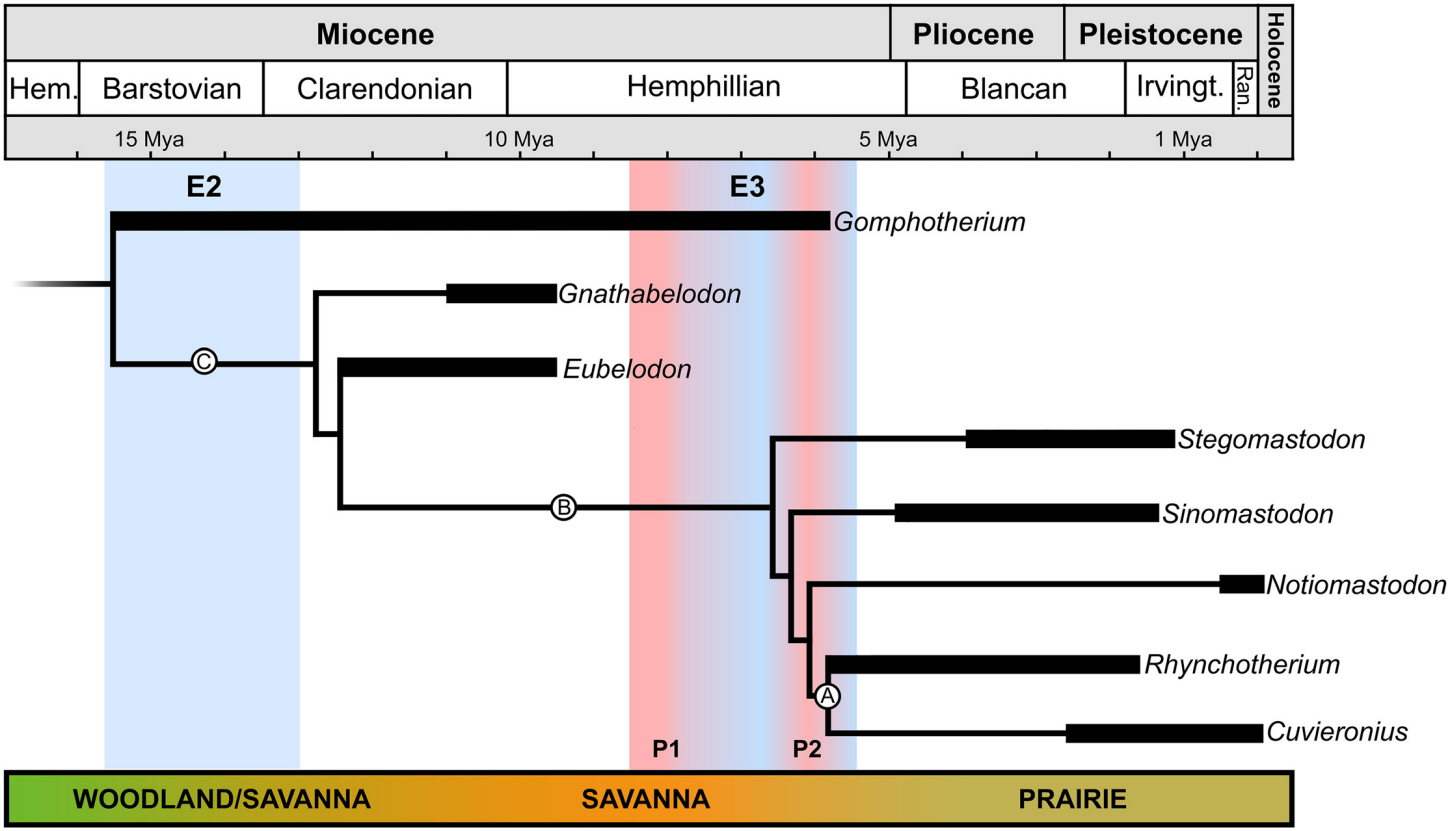
Phylogenetic position of *Cuvieronius hyodon*. Clade A includes *Rhynchotherium* and *Cuvieronius*, Clade B includes all brevirostrine non-amebelodontine trilophodont gomphotheres and Clade C represents the ingroup analyzed in this study. E2 and E3 represent the most abrupt climatic changes during Neogene, shades of blue represent decrease and shades of red represent increase in temperature and environmental humidity. The biome transition bar represent the vegetation changes during Neogene to North America.

To better understand the “dance” of tusks in the non-amebelodontine trilophodont gomphotheres, we conducted a phylogenetic revision of this group, with especial emphasis on the addition of new characters related to lower incisors. Our phylogenetic analysis resulted in one most parsimonious tree (32 steps; CI = 0.781, RI = 0.8) and recovered *Cuvieronius hyodon* and *Rhynchotherium falconeri* as sister-taxa (Clade A in Fig 3). This clade is defined by seven unambiguous synapomorphies: the presence of an enamel band in the upper tusks (character 1, state 2); twisted upper tusks (character 3, state 1); presence of lower tushes (character 4, state 0); presence of the incisors fossa (character 11, state 0); ventral torsion of mandible symphysis ≥35° (character 13, state 1); oval/circular cross section of lower tushes (character 16, state 1); and upper tusks alveoli greatly diverging distally (character 19, state 2).

We are not the first to suggest the sister-taxa relationship between *C*. *hyodon* and *R*. *falconeri*. An almost complete skull and mandible of *Rhynchotherium* from Arizona (USA) was described [40] and the author suggested *Rhynchotherium* as a precursor of *Cuvieronius*. Likewise, a close relationship was stated between both taxa [31], based on the spiral enamel bands on the upper tusks and brevirostrine mandibles, although the authors did not perform a formal cladistic analysis to test this hypothesis. Additionally, these authors indicated that the presence of lower incisors and downturned symphysis distinguish *R*. *falconeri* from *C*. *hyodon*. We disagree with their interpretation of about both features, because *C*. *hyodon* also presents lower incisors (in juvenile individuals from J1 to J5 developmental stages) and the mandibular symphysis in *C*. *hyodon* is as much downturned as it is in *R*. *falconeri* (35° or more). However, we identified differences between *C*. *hyodon* and *R*. *falconeri* in this study; such as *R*. *falconeri* having a longer symphysis than *C*. *hyodon* (an exceptional condition among brevirostrine gomphotheres), which accommodates robust lower tushes. The lower tushes of *R*. *falconeri* are slightly upcurved and with divergent tips (Fig 2B), whereas *C*. *hyodon* had slender, straight and parallel to convergent incisors (Figs 1B and 2A). In addition, both lower tushes have open pulp cavity, an evidence of an evergrowing condition, however, *Cuvieronius* has a delayed loss of the lower tushes (J5 developmental stage), compared to *Gomphotherium* (J1 developmental stage), while *Rhynchotherium* retained the evergrowing lower tushes during all lifespan.

Here, we provide the first phylogenetic hypothesis that take into consideration the presence of lower tushes in *Cuvieronius hyodon*. Furthermore, this is the first analysis in which *Cuvieronius* and *Rhynchotherium* were found as a natural group (Clade A, Fig 3), supported by the presence of the lower tushes and six other unambiguous synapomorphies. Additionally, if we consider the presence of lower tushes in *Cuvieronius* in previously published phylogenies [1,5,6,21,22] this feature is inferred to have occurred three times, independently, within the non-amebelodontine trilophodont gomphotheres (in *Gomphotherium*, *Rhynchotherium* and *Cuvieronius*). In the present study, the presence of lower tushes occurs only two times, independently, in *Gomphotherium* and in Clade A (Fig 3). It is important to highlight that, probably, the ancestor of the Clade A had lower tushes, at 6 Mya (Fig 3), and this feature may be a case of evolutionary reversal, because the lower tushes were lost (apomorphic/derived condition) at the base of the Clade C (Fig 3).

**Fig 4:**
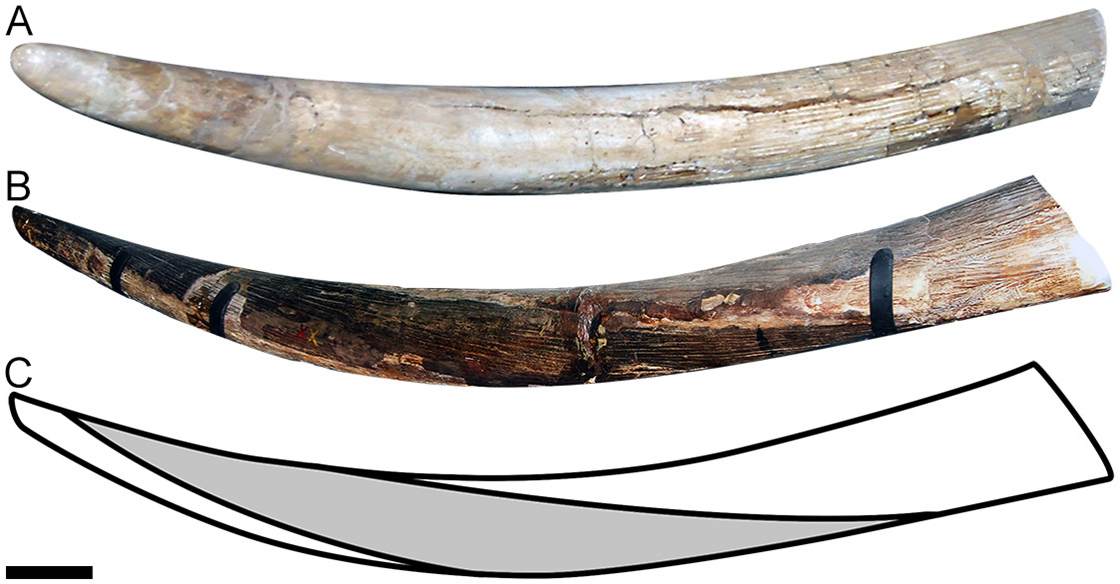
Upper tusks morphotypes of Clade B. (A) Not twisted upper tusk of *Notiomastodon platensis*, specimen MLP-8-1, from Arrecifes, Argentina; (B) Twisted upper tusk of *Cuvieronius hyodon*, specimen MACN-Pv 1891, from Tarija, Bolivia; (C) Schematic drawing of twisted enamel band of specimen represented in B, the grey area represents the twisted enamel band. Scale bar = 10 cm.

Another synapomorphy of Clade A related to the incisors is the presence of a pair of twisted upper tusks (Fig 4), which is also an exclusive and unique condition within the order Proboscidea [6,29]. The twisted upper tusks of *Rhynchotherium falconeri* and *Cuvieronius hyodon* (I2) are usually long, straight to slightly upcurved in lateral view, and symmetric (they mirror each other, in right-hand torsion, Fig 4). The growth of naturally twisted structures is usually interpreted as a associated response to environmental stresses. Besides, such structures are mechanically advantageous, enhancing their impact resistance and fracture toughness. In addition, in a study on narwhal tusks, the spiral mode of growth of the tooth ensures overall straightness of the incisor, even if it grows irregularly [41]. In this way, the same process was probably positively selected in Clade A, as a response to a demand for long and resistant upper tusks. It is important to highlight that the upper tusks of *Rhynchotherium* and *Cuvieronius* are not spiraled to the same extent (the enamel band in the former seems to present more turns around the ivory than in the latter), and therefore *Rhynchotherium* probably presents upper tusks that are more resistant to mechanical stress than *Cuvieronius*. However, as we did not evaluate a large sample so far, the level of spiralization in the enamel band of upper tusks could also be individually or ontogenetically variable in both taxa.

### The “dance” of tusks in *Cuvieronius hyodon* and its closest partners

An intense and fast aridification, along with cooling climatic changes occurred during the middle-late Miocene, Early Barstovian to Early Clarendonian (E2, Fig 3; [42]) in North America. Especially in the Southwest United States and the Great Plains, this drastic climatic shift also promoted an environmental change from Woodland to Savanna [43], which also possibly led to the loss of lower tusks in the Clade C (Fig 3). At some point between the early Clarendonian and the middle-late Hemphillian, the North American Savanna was the scenario for the origin and diversification of Clade B (Fig 3). This clade includes all New World non-amebelodontine trilophodont gomphotheres (*Stegomastodon*, *Notiomastodon*, *Cuvieronius* and *Rhynchotherium*) and the Asian *Sinomastodon*. Clade B is diagnosed by four unambiguous synapomorphies: the upper tusks upturned, absence of lower incisors, brevirostrine mandible and upper tusk alveoli slightly diverging. The brevirostrine condition of mandible occurred independently of (and later than) the loss of lower incisors (Fig 3). The association of longirostrine and tuskless mandibles is very rare in proboscideans, and only *Choerolophodon* (family Choerolophodontidae, *sensu* [23]), a recently described new genus of Mammutidae [44], as well as the non-amebelodontine trilophodont gomphotheres *Gnathabelodon* and *Eubelodon* show these two conditions simultaneously. Moreover, the emergence of brevirostry in Clade B appears to be related to a more open environment, the Savanna (Fig 3), which is in accordance with previous studies [6].

The presence of lower tushes is an unambiguous synapomorphy of Clade A, and it can be explained by the evolutionary process of taxic atavism [38,39,45,46]. This evolutionary process refers to the reappearance of a character state typical of a remote ancestor (in this case, the presence of lower tushes, the symplesiomorphic state in *Gomphotherium*) in a later deriving lineage (the common ancestor of Clade A). Taxic atavism is very rare in vertebrates [38,46]. Accordingly, the retrieval of the lower tushes in the ancestor of the Clade A probably occurred at 6 Mya (middle to late Hemphillian, Fig 3) at the Great Plains of North America [43] and it is a unique case within Elephantimorpha (*sensu* [23], Fig 3). The second peak of warmth and humidity (P2) at the E3 climatic event affected the Great Plains [47], and this climatic event is associated herein to the reacquisition of lower tushes in Clade A (Fig 3). However, after this warming period, the environmental humidity and temperature levels rapidly and drastically decreased again, allowing for the domination of C_4_ grasses in the Great Plains grasslands [48].

Therefore, due to this fast climatic change, when environment conditions became cooler and dryer, the evolution of lower tushes followed different paths in *Rhynchotherium falconeri* and *Cuvieronius hyodon*. The ontogenetic process that explains such structure in Clade A could be the Neotenic Paedomorphism, in which a feature grows in a slower velocity (heterochrony) when compared with the velocities of growth in an ancestor. *Rhynchotherium* could represent the full process of paedomorphism, since it retained the evergrowing lower tushes (di1) during all lifespan. *Cuvieronius*, in turn, presented a shorter period of retention of the lower tushes (typical juvenile structure), until an older developmental stage (J5; since *Gomphotherium* lost the di1 in Fetus/J1 developmental stages). Moreover, the pedomorphic neoteny is more evident in *Rhynchotherium* because, by definition, it generates adults that resemble or, in this case, bear juvenile features of their ancestors (presence of di1).

### The “dance” of tusks in Elephantimorpha

**Fig 5:**
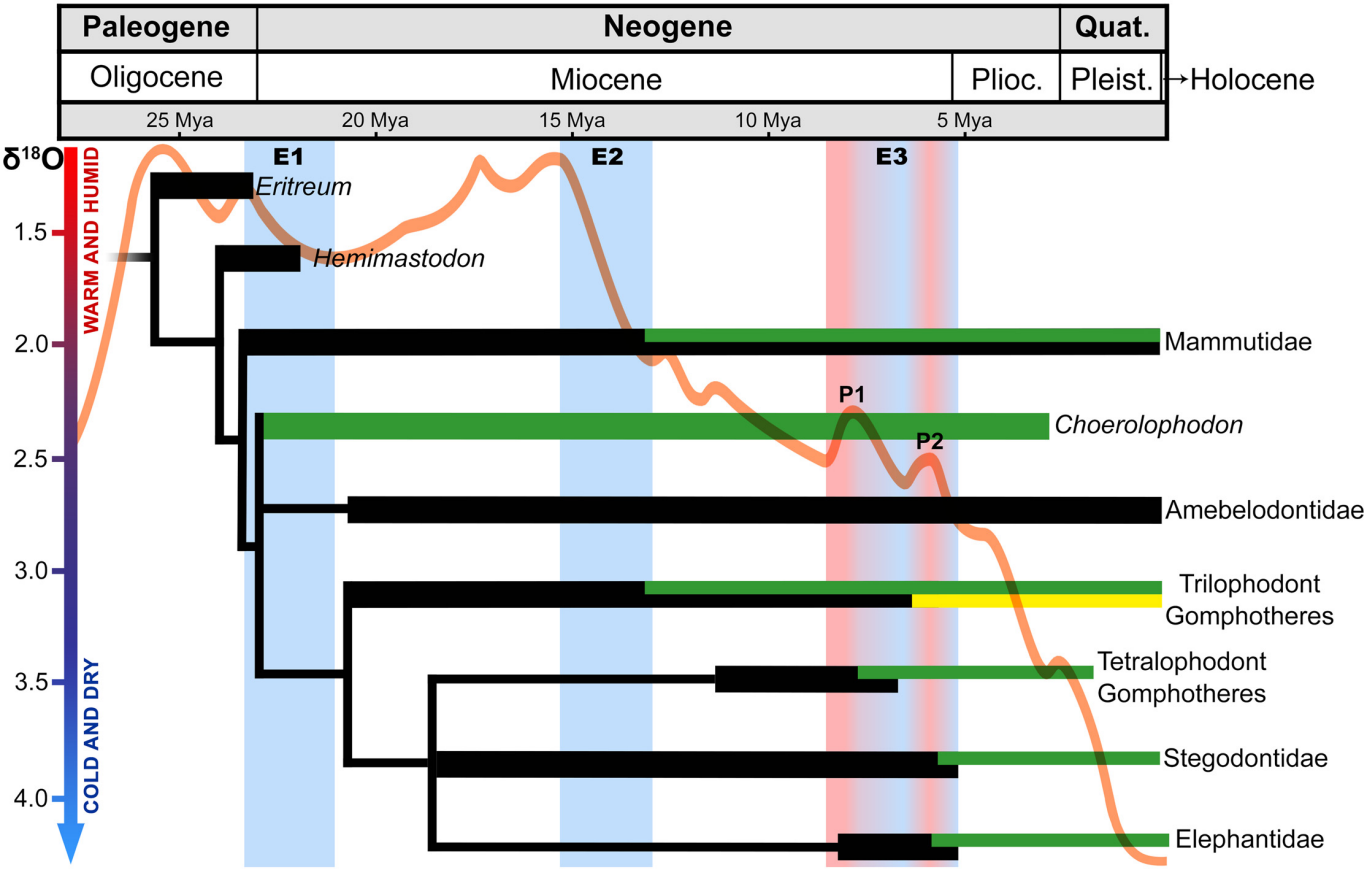
Time-calibrated phylogenetic relationship of Elephantimorpha in association to climatic fluctuations from Paleogene to Quaternary. The phylogeny was modified from [23,49]. Black bars represent tetralophodont proboscideans; green bars represent dibelodont proboscideans and yellow bar represents tetrabelodont proboscideans (reversion/atavism). The orange line represents temperature and humidity variations according to [47,50]. E1, E2 and E3 represent the most abrupt climatic changes, shades of blue represents decrease and shades of red represents increase in temperature and environmental humidity.

*Cuvieronius hyodon* was recovered as a derived gomphothere in most previous phylogenetic hypotheses [1,5,6,21,22]. This position is extremely interesting, since *Cuvieronius* had lower tushes, a condition usually considered as plesiomorphic for Proboscidea [6]. Moreover, the tetrabelodont condition is also considered plesiomorphic to Elephantimorpha. The loss of lower incisors is recorded in most lineages of Elephantimorpha, and only a few (Mammutidae, Amebelodontidae and non-amebelodontine trilophodont gomphotheres) retained lower incisors after the Miocene (Fig 5). Accordingly, we believe that the loss of lower incisors in Elephantimorpha occurred independently, in three occasions during the Miocene (E1, E2 and E3, Fig 5).

Therefore, what drove the negative selection of lower incisors in most lineages of Elephantimorpha? This natural group of Proboscidea was distributed throughout all continents from the Late Oligocene to the Pliocene/Pleistocene transition (except in Antarctica, Australia and South America; [6]) and only two taxa of non-amebelodontine trilophodont gomphotheres were recorded in South America during Pleistocene [3,14]. Here, we infer that due to the wide geographic range of Elephantimorpha during Miocene, the independent negative selection of its lower incisors may have been caused by global scale climatic events. Accordingly, the Miocene epoch is known by its climatic instability [50] and climatic fluctuations are one of the best candidates to promote such evolutionary selections (Fig 5). The climatic variations in global temperature and humidity throughout the Miocene, the "Zachos Curve” [50], is compared to a time-calibrated phylogenetic hypothesis for Elephantimorpha [6,23,44,49], most of the rapid cooling/drought events (E1, E2 and E3) coincides with the loss of lower incisors in distinct lineages of Elephantimorpha (Fig 5).

However, how did these climatic events negatively select the lower incisors in most lineages of Elephantimorpha? The highest heat transfer in Proboscidea occurs in the ears, trunk and incisors tips [51,52]. Thus, in a colder climatic condition, a tetrabelodont proboscidean (with four incisors: a lower and an upper pairs) would lose more heat to the environment than a dibelodont proboscidean (with only the upper pair of incisors). In addition, the heat conductivity of proboscidean ivory is low, mainly when compared to isolated dentine and enamel [53]. Thus, incisors with large amount of enamel should also be responsible for a higher heat loss than incisors with a smaller amount of enamel (an enamel tip or small band).

Furthermore, the incisors of proboscideans have several important roles on their biology, including feeding and mating [54] and probably, because of these functions, the natural selection did not act negatively on both upper and lower incisors and positively preserved only one pair; in the case of Elephantimorpha, the upper one. In addition, we also infer that the loss of the enamel in tusks and the loss of lower incisors may be a response to drastic decrease in global temperature and humidity, as a way to preserve body thermal energy in a cold and dry environment.

The E1 climatic shift, which we infer as the one driving the negative selection of the lower tusks in Elephatimorpha, occurred at the transition Paleogene/Neogene (approximately 23.3 to 21 Mya) and comprised the loss of lower incisors in the most recent ancestor to *Choerolophodon* and other lineages of Elephantimorpha (Fig 5). Subsequently, Earth experienced a period of gradual increase in global temperatures and humidity, reaching the apex of these conditions around 17 to 15 Mya, a period known as the middle Miocene Climatic Optimum [55]. The Miocene Climatic Optimum was followed by E2 (approximately 15 to 13 Mya), which is characterized by an abrupt decrease in global temperatures and humidity.

The second climatic event is marked by the radiation of dibelodont forms in two lineages of Elephantimorpha: the Mammutidae and the non-amebelodontine trilophodont gomphotheres (green bars in Fig 5), around 13.5 Mya. The loss of lower incisors occurred simultaneously in these two lineages, probably as a response to the colder and dryer climatic conditions after the Miocene Climatic Optimum. The decrease in global temperatures and humidity was less abrupt after the E2, but persisted until 8.5 Mya (Fig 5), when it was interrupted by E3 (the third and last climatic event). Broadly speaking, the E3 was a period of slow global cooling and drought. However, it also comprised two abrupt peaks of warmth and humidity (P1 and P2; Fig 5). No significant event in the evolution of Elephantimorpha occurred during the ascending of P1, but at the decline from P1 (cooling and drought climatic change) occurred the rise of dibelodont forms in tetralophodont gomphotheres (green bar in Fig 5), followed by the extinction of its tetrabelodont forms at the end of P1 (black bar in Fig 5).

The ascending to P2 is marked by the rise of tetrabelodont forms in the non-amebelodontine trilophodont gomphotheres, throughout the recovery of lower tushes in the common ancestor of the *Rhynchotherium*-*Cuvieronius* clade (clade A in Fig 5) after the loss of these structures at the origin of Clade C (Fig 4), which occurred in E2 (the yellow bar in Fig 5), probably as a response to the increasing of global temperature and humidity. Several evolutionary events occurred at the decline of P2, as the extinction of the tetrabelodont trilophodont gomphothere *Gomphotherium* (the black bar in Fig 5), the rise of dibelodont forms (green bars in Fig 5) of Stegodontidae and Elephantidae, and the extinction of the tetralophodont stegodontids and elephantids at the end of P2. After E3, the vertiginous increase of cold and dry climatic conditions was recorded, and all derived lineages of Elephantimorpha became extinct during the Pleistocene or at the Pleistocene/Holocene boundary, except for the dibelodont extant elephants *Loxodonta* and *Elephas* (Elephantidae; [6]).

Among Elephantimorpha, *Cuvieronius hyodon* is the unique tetrabelodont non-amebelodontine trilophodont gomphothere to reach the latest Pleistocene (INAH uncatalogued specimen, [27]). Nonetheless, to understand the “dance” of incisors in the non-amebelodontine trilophodont gomphotheres, the presence of lower tushes in *Cuvieronius hyodon* must be scored in a phylogenetic context.

## Conclusion

We confirmed here the presence of vestigial lower tushes in *Cuvieronius hyodon*, which probably were in use from all juvenile stages (J1–J5) to the first of the young stage (Y1). The presence of lower tushes is one of the seven synapomorphies that diagnoses the well-supported clade including *Cuvieronius hyodon* and *Rhynchotherium falconeri*. Moreover, we recognized that the reappearance of lower tushes in this clade is an evolutionary process of taxic atavism in Proboscidea, and a case of pedomorphic neoteny, since adult *Rhynchotherium* presented these “milk and juvenile” structures.

The loss of enamel in incisors and the loss of lower incisors in all lineages of Elephantimorpha is probably a result of a negative selection related to the decrease of global temperature and humidity, occurred after the Miocene Climatic Optimum (26 to 15 Mya) on. The recovering of the lower tushes by the clade including *Cuvieronius hyodon* and *Rhynchotherium falconeri* is an unique case of taxic atavism within the Elephantimorpha, and is possibly related to an increasing apex of temperature and humidity during the Late Miocene (around 6 Mya) at the Great Plains. In addition, the twisted upper tusks of *Rhynchotherium* and *Cuvieronius* is also an exclusive synapomorphy of this clade and probably occurred in response to the need of long and resistant upper incisors.

## Materials and Methods

We analyzed 46 mandibles of *Cuvieronius hyodon*, including young and adult individuals from Tarija (Bolivia), Mexico, Costa Rica and United States (Table 1). All mandibles analyzed in this study were previously identified as *Cuvieronius hyodon* through the association of diagnostic materials, such as upper tusks and/or skulls [3]. The studied specimens are housed in the paleontological collections of Museo de Ciencias Naturales ‘Bernardino Rivadavia’ (MACN-Pv), Argentina; Museo Nacional de Paleontología y Arqueología de Tarija (MNPA or TAR), Bolivia; American Museum of Natural History (AMNH-DVP, F:A.M.), United States of America; Escuela Centroamericana de Geología de la Universidad de Costa Rica (UCR), Costa Rica; Instituto Nacional de Antropología y Historia (INAH) and Centro de Geociencias de la Universidade Nacional Autónoma de México, Campus Juriquilla, Mexico; Swedish Museum of Natural History, Sweden (NMR); and Muséum National d’Histoire Naturelle, France.

Each mandible was assigned to a developmental stage based on the functional teeth under usage before death and their stages of wearing (modified from [7,8,20]). The developmental stages for *Mammut americanum* were based on pre-molars and molars eruption and wear stages, from "Fetus" to "Young adult” (total of nine developmental stages, [7]), while others 23 dental age classes were suggested to *Gomphotherium angustidens* [30]. However, comparisons across Gomphotheriidae and Mammutidae indicate their series of teeth wear patterns may differ, since *G*. *angustidens* presents permanent pre-molars and subsequent teeth usually differ from one to two wear stages in brevirostrine gomphotheres [8], which does not happen in *Mammut*. In this way, we adapted the wear stages proposed to *Mammut* and *Gomphotherium* to best fit the developmental stages identified in *Cuvieronius hyodon* (total of eleven developmental stages; see Table 1), by changing the developmental stage "Young adult” to Y3 and proposing the developmental stages Y4 (dp4 in severe wear and m1 in wear stage 2) and Adult (only permanent molars in use).

Moreover, we analyzed the presence of lower incisors and/or lower incisors alveoli in each mandible, with the alveoli being classified into two categories [11]: open alveoli (a pair or a single cylindrical cavity, usually symmetric and posteriorly directed) or closing alveoli (a pair or a single oval depression, usually symmetric, filled with spongy tissue). The morphological features of *Cuvieronius hyodon* lower incisors were described and compared to those of immature individuals of *Rhynchotherium falconeri* [31] and *Gomphotherium angustidens* [9,28,30].

We performed a phylogenetic analysis in order to elucidate the relationships of *Cuvieronius hyodon* within the non-amebelodontine trilophodont gomphotheres (*sensu* [1]). In this study, we use this term instead of trilophodont gomphotheres to represent the lineage inclusive of *Gomphotherium*, *Rhynchotherium*, *Gnathabelodon*, *Eubelodon*, *Stegomastodon*, *Cuvieronius*, *Notiomastodon* and *Sinomastodon* [6]. We propose a new data matrix of twenty-four dental, mandible and cranial characters (data in S1 Appendix). Our dataset consists of characters modified from the literature [1,6,21] as well as new characters, such as the downward deflection of the mandibular symphysis and the difference in height between the mandibular condyle and the coronoid process (data in S1 Appendix). We run the data matrix in the software TNT [56], using the exact search algorithm “implicit enumeration” and characters with equal weights. The outgroup is represented by *Gomphotherium angustidens*, and the ingroup includes *Eubelodon morrilli*, *Gnathabelodon thorpei*, *Rhynchotherium falconeri*, *Cuvieronius hyodon*, *Stegomastodon* (only North American species, see [3]), *Notiomastodon platensis* (= *Haplomastodon chimborazi*, “*Stegomastodon*” *waringi*, “*Stegomastodon*” *platensis*) and the Asian taxon *Sinomastodon*, as it is commonly considered closely related to New World gomphotheres [56]. However, we did not include *Amahuacatherium* and *Megabelodon* in the present analysis. Whereas *Amahuacatherium* has been previously considered to be an invalid genus [3], *Megabelodon* is probably part of *Gomphotherium* [6], a position which we agree with. We considered all species of *Stegomastodon* (North American species [2]) and *Sinomastodon* [57] during character-state scoring because the species diversity of these taxa is still under revision.

## Supporting Information

S1 Appendix Characters list and Data matrix of non-amebelodontine trilophodont gomphotheres. (DOCX)
