# Supplementary material for: The Dance of Tusks: Rediscovery of Lower Incisors in the Pan-American Proboscidean Cuvieronius hyodon Revises Incisor Evolution in Elephantimorpha
Source: PLoS One. 2016 Jan 12;11(1):e0147009. doi: 10.1371/journal.pone.0147009 (PMC4710528; doi:10.1371/journal.pone.0147009)
Supplement: S1 Appendix — (DOCX) [file pone.0147009.s001.docx]

**Supporting Information**

**S1 Appendix. Characters list and Data matrix of non-amebelodontine trilophodont gomphotheres.**

1. Enamel in I2: absent (0), present covering partially or totally the incisor (1), present as a band (2);
2. I2 in lateral view: downturned (0), straight (1), upturned (2);
3. I2: not twisted (0), twisted (1);
4. di1: present (0), absent (1);
5. i1: present (0), absent (1);
6. Obliquity in dp3 posterior loph: oblique (0), not oblique (1);
7. M3 postentoconule: M3 is trilophodont (0), M3 is tetralophodont (1), M3 is pentalophodont (2), M3 is hexalophodont or more (3);
8. Molar trefoils: absent (0), on pretrites (1), on pretrites and posttrites (2);
9. m3/M3 medial cingulum: absent (0), present (1);
10. Molar main cusp and mesoconelet: unequal in size (0), equal in size (1);
11. Incisors fossa on premaxillary: present (0), absent (1);
12. Mandibular symphysis: longirostrine (0), brevirostrine (1);
13. Ventral torsion of mandible symphysis: less than 35º (0), more than 35º (1);
14. Mandible angle process: prominent (0), reduced (1);
15. Coronoid process and condyle heights: condyle slightly above coronoid process (0), condyle far above coronoid process (1);
16. di1 cross-section: piriform/flat (0), oval/circular (1);
17. Forehead: narrow (0), wide (1)
18. Premaxillaries in frontal view: slender, distal margin much narrower than interorbital width (0), robust, distal margin equal or wider than interorbital width (1);
19. Upper tusk alveoli (in frontal view): nearly parallel (0), distally slightly diverging (1), distally greatly diverging (2);
20. Mandibular
21. ascending ramus: dorso-posteriorly directed (0), dorsally directed (1);Supraorbital foramen: present (0), absent (1);
22. Subnasal fossa: absent (0), present (1);
23. Enamel in di1: present covering partially or totally the incisor (0), present as a band (1);
24. di1 in superior view: parallel to convergent (0); parallel to divergent (1).

| Taxa | 1 | 2 | 3 | 4 | 5 | 6 | 7 | 8 | 9 | 10 | 11 | 12 | 13 | 14 | 15 | 16 | 17 | 18 | 19 | 20 | 21 | 22 | 23 | 24 |
| --- | --- | --- | --- | --- | --- | --- | --- | --- | --- | --- | --- | --- | --- | --- | --- | --- | --- | --- | --- | --- | --- | --- | --- | --- |
| *Gomphotherium* | [02] | 0 | 0 | 0 | 0 | 0 | [01] | [01] | 0 | 0 | 1 | 0 | 0 | 0 | 0 | 0 | 0 | 0 | 0 | 0 | 0 | 0 | 0 | 0 |
| *Cuvieronius* | 2 | 2 | 1 | 0 | 1 | 1 | [12] | [12] | 1 | 1 | 0 | 1 | 1 | 1 | 1 | 1 | 1 | 1 | 2 | 1 | 1 | 1 | 0 | 0 |
| *Rhynchotherium* | 2 | 2 | 1 | 0 | 1 | 1 | 1 | 1 | 1 | 1 | 0 | 1 | 1 | 1 | 1 | 1 | 1 | 1 | 2 | 1 | 1 | 1 | 1 | 1 |
| *Notiomastodon* | [012] | [12] | 0 | 1 | 1 | 1 | [12] | [12] | 1 | 1 | 1 | 1 | 0 | 1 | 1 | ? | 1 | 1 | 1 | 1 | 1 | 1 | ? | ? |
| *Stegomastodon* | 0 | [12] | 0 | 1 | 1 | ? | [23] | 2 | 1 | 0 | 1 | 1 | 0 | 0 | 0 | ? | 1 | 0 | 1 | 0 | 0 | 1 | ? | ? |
| *Sinomastodon* | 0 | 2 | 0 | 1 | 1 | 1 | 2 | 1 | 1 | 0 | ? | 1 | 0 | 0 | 0 | ? | 1 | 1 | 1 | 1 | 0 | 0 | ? | ? |
| *Eubelodon* | 2 | 0 | 0 | ? | 1 | ? | 1 | 2 | 1 | 0 | 0 | 0 | 0 | 0 | 0 | ? | 1 | 0 | 0 | 0 | 1 | 1 | ? | ? |
| *Gnathabelodon* | 0 | 0 | 0 | ? | 1 | ? | 1 | 1 | 0 | 0 | 1 | 0 | 0 | 0 | 0 | ? | ? | 0 | 0 | 0 | 0 | 0 | ? | ? |
